# Prediction of the risk of developing hepatocellular carcinoma in health screening examinees: a Korean cohort study

**DOI:** 10.1186/s12885-021-08498-w

**Published:** 2021-06-29

**Authors:** Chansik An, Jong Won Choi, Hyung Soon Lee, Hyunsun Lim, Seok Jong Ryu, Jung Hyun Chang, Hyun Cheol Oh

**Affiliations:** 1 Department of Radiology, National Health Insurance Service Ilsan Hospital, Goyang, South Korea; 2 Research Institute, National Health Insurance Service Ilsan Hospital, Goyang, South Korea; 3 Department of Internal Medicine, National Health Insurance Service Ilsan Hospital, Goyang, South Korea; 4 Department of Surgery, National Health Insurance Service Ilsan Hospital, Goyang, South Korea; 5 Department of Otolaryngology-Head and Neck Surgery, National Health Insurance Service Ilsan Hospital, Goyang, South Korea; 6 Department of Orthopedic Surgery, National Health Insurance Service Ilsan Hospital, Goyang, South Korea

**Keywords:** Big data, Machine learning, Liver neoplasms, Precision medicine

## Abstract

**Background:**

Almost all Koreans are covered by mandatory national health insurance and are required to undergo health screening at least once every 2 years. We aimed to develop a machine learning model to predict the risk of developing hepatocellular carcinoma (HCC) based on the screening results and insurance claim data.

**Methods:**

The National Health Insurance Service-National Health Screening database was used for this study (NHIS-2020-2-146). Our study cohort consisted of 417,346 health screening examinees between 2004 and 2007 without cancer history, which was split into training and test cohorts by the examination date, before or after 2005. Robust predictors were selected using Cox proportional hazard regression with 1000 different bootstrapped datasets. Random forest and extreme gradient boosting algorithms were used to develop a prediction model for the 9-year risk of HCC development after screening. After optimizing a prediction model via cross validation in the training cohort, the model was validated in the test cohort.

**Results:**

Of the total examinees, 0.5% (1799/331,694) and 0.4% (390/85,652) in the training cohort and the test cohort were diagnosed with HCC, respectively. Of the selected predictors, older age, male sex, obesity, abnormal liver function tests, the family history of chronic liver disease, and underlying chronic liver disease, chronic hepatitis virus or human immunodeficiency virus infection, and diabetes mellitus were associated with increased risk, whereas higher income, elevated total cholesterol, and underlying dyslipidemia or schizophrenic/delusional disorders were associated with decreased risk of HCC development (*p* < 0.001). In the test, our model showed good discrimination and calibration. The *C*-index, AUC, and Brier skill score were 0.857, 0.873, and 0.078, respectively.

**Conclusions:**

Machine learning-based model could be used to predict the risk of HCC development based on the health screening examination results and claim data.

**Supplementary Information:**

The online version contains supplementary material available at 10.1186/s12885-021-08498-w.

## Background

Hepatocellular carcinoma (HCC) is the third most common cause of cancer death worldwide, with over half a million new cases diagnosed annually worldwide [1, 2]. In South Korea (hereafter Korea), HCC and other primary liver cancer are the fourth most common cancer in men and the sixth in women, and the second largest cause of cancer mortality [3].

Almost all Koreans are covered by mandatory national health insurance or Medical Care (a governmental program corresponding to the US Medicaid), and all insured adults aged 40 years or older are required to undergo a national general health screening examination at least once every 2 years. All the claim and health screening data produced are accumulated in the database of the national health insurance system and can be used for a research purpose with permission. The national health screening examination is intended for screening general health risk factors. However, we postulated that new values could be derived that can be used to predict the risk of development of a certain disease if the examination results are used in combination with the claim data.

As the healthcare insurance claim and screening data contain information related to the risk of developing HCC such as demographic characteristics, family medical history, laboratory results including liver enzymes, and various underlying medical conditions including chronic liver disease and viral infection [4], we hypothesized that a machine learning algorithm may be utilized to predict the risk of HCC for each participant of the national health screening examination.

Several models have been proposed to predict the risk of HCC development [5–12]. However, to the best of our knowledge, most of them were for patients who are already at high risk for HCC. If a prediction model targets all screening examinees that include not only people who are already aware of their risks for HCC but also those who are not, it could play an additional important role in identifying undiagnosed high-risk patients.

Therefore, the purpose of this study was to identify risk factors and develop a machine learning model to predict the risk of HCC development for an individual examinee within 9 years after the national health screening examination with a large cohort of Koreans.

## Methods

### Study population

The National Health Insurance Service-National Health Screening (NHIS-HEALS) database is a sample cohort of 514,795 people, accounting for 10% of all health screening examinees aged 40–80 years in 2002 or 2003 in South Korea, and contains the information on their claim data and the results of their health screening examinations between 2002 and 2015. Detailed information on the NHIS-HEALS database has been outlined elsewhere [13, 14]. This retrospective cohort study was approved by the Institutional Review Board of National Health Insurance Service Ilsan Hospital (NHIMC 2020–06-033), and the informed consent from the participants was waived.

**Fig. 1:**
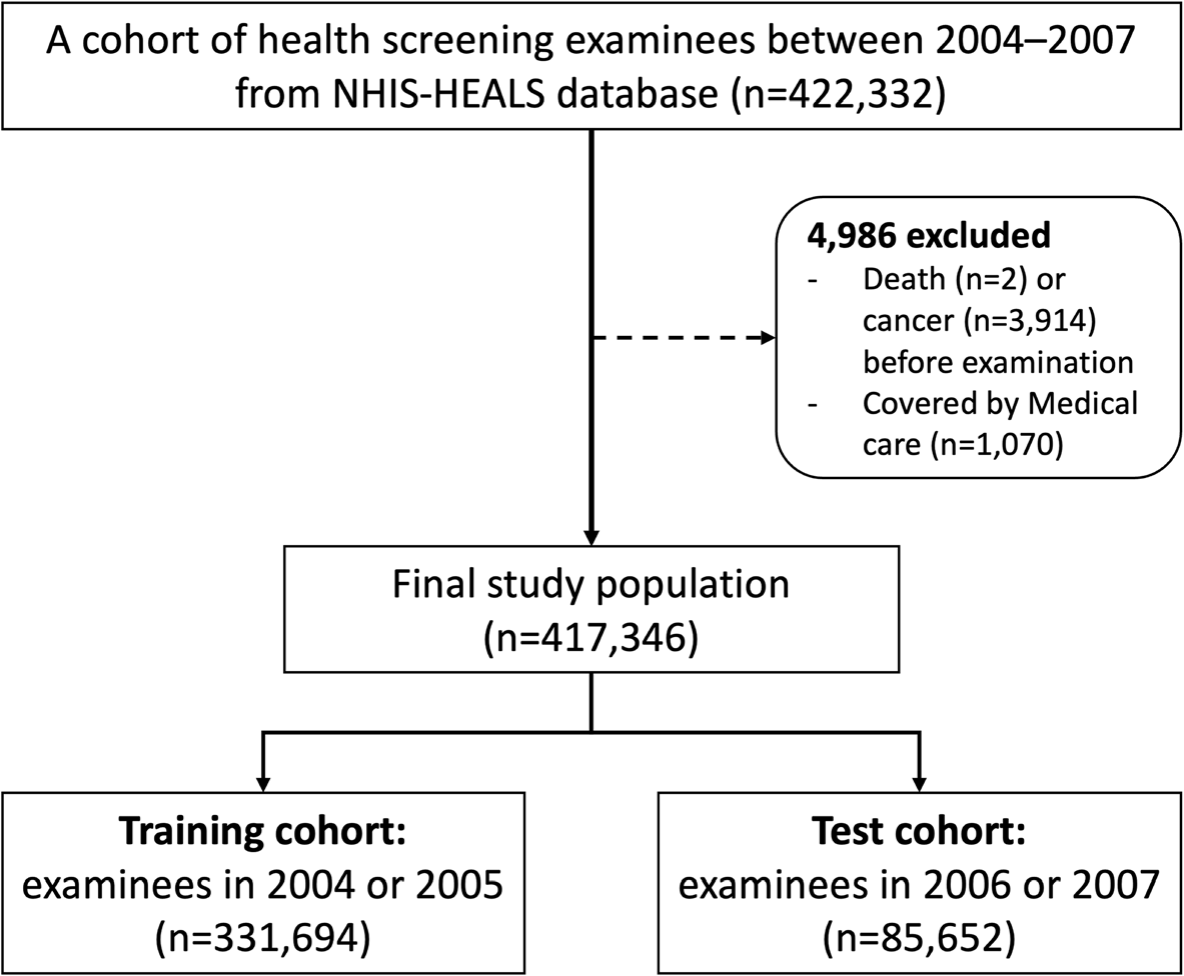
Study flow chart

Of the total 514,795 people, 334,966 who also underwent the health screening in 2004 or 2005 were included in a training cohort, with 2002 and 2003 used as a washout period. In addition, of the remaining 179,829 people, 87,416 who underwent the health screening in 2006 or 2007 (but not 2004 or 2005) were identified, and this cohort was used as a test cohort, with the years before 2006 used as a washout period. People who died (*n* = 2) or were diagnosed as having cancer (*n* = 3914) during the washout period were excluded. Furthermore, people covered by Medical Care (*n* = 1070) were excluded because their healthcare service claim is significantly different than the general population. The ratio of the training cohort to the test cohort was approximately 8:2 (Fig. 1). The ratio of 8:2 is commonly used as a rule-of- thumb when splitting a dataset into training and test sets; a recent machine-learning study also reported that using 70% or 80% of the data as a training set showed the best result [15].

### Variables

Input and outcome variables were extracted from the NHIS-HEALS cohort following processing and cleaning the data. The full description of variables included can be found in Supplementary Table 1.

#### Input variables

Variables retrieved from the healthcare claim data included sociodemographic variables, underlying medical conditions, and prescription records. In the NHIS-HEALS cohort, diagnoses were coded according to the Korean Standard Classification of Diseases 6th revision (KCD-6) [16], which is based on the International Classification of Diseases 10th revision (ICD-10) [17]. However, the diagnosis claimed by the healthcare providers and the actual diagnosis may differ because the dataset was established for recording claims and reimbursements. Therefore, for major diseases such as hypertension, diabetes mellitus (DM), dyslipidemia, heart diseases, and stroke, operational definition was used as previously reported [18]. For example, the diagnosis of hypertension was determined to occur when a patient on antihypertensive medication was admitted for the first time or visited outpatient clinic for a second time with ICD-10 codes for hypertensive disease. (See Supplementary Table 2 for the definitions of all the underlying medical conditions used in this study). In the NHIS-HEALS data, some diagnostic codes were masked as sensitive personal information; for example, human immunodeficiency viruses (HIVs) (B20–B24) were grouped under the B_ code, and mental and behavioral disorders due to psychoactive substance use (F10–F19) and schizophrenia, schizotypal and delusional disorders (F20–F29) were coded as F_ altogether.

The health screening data included physical examination results (height, weight, and blood pressure), laboratory results (fasting glucose, total cholesterol, hemoglobin, urine stick test, liver enzymes), information obtained from history taking or questionnaires (family medical history, smoking history, alcohol consumption, and exercise habit).

#### Outcome variables

For probability prediction (i.e., classification task), the outcome was whether HCC was diagnosed within 9 years from the health screening examination. For time-to-event prediction (i.e., survival analysis), the outcome was the time interval between the examination and the diagnosis. The diagnoses of other cancers were considered competing risks. Participants who were not diagnosed with HCC until the last follow-up date or who died from other causes during the follow-up period were right-censored, meaning that the survival time is incomplete at the right side of the follow-up period. The NHIS-HEALS data contains the date and cause of death statistics extracted from the national database produced by Statistics Korea. The last follow-up date was December 31, 2015.

### Statistical analysis and machine learning

All analyses were performed using R 3.3.3. Main packages used include ‘survival (v2.41–3)’, ‘cmprsk (v2.2–7)’, ‘randomForestSRC (v2.5.1)’, ‘caret (v6.0–78)’, ‘survminer (0.4.2)’, and ‘xgboost (0.6.4.1)’. In Table 1, continuous and categorical variables were compared using Mann-Whitney or t-test and chi-square test, respectively. Continuous variables were expressed as mean with standard deviation. Two-sided probability values of < 0.05 were considered statistically significant.

**Table 1:** Baseline characteristics in the training and test cohorts

Variable		Training cohort (*n* = 331,694)	Test cohort (*n* = 85,652)	*p*-value	Total (*n* = 417,346)
**Sociodemographic characteristics**					
Age (years)		54.3 (9.28)	57.7 (9.6)	< 0.001	55.0 (9.45)
Sex	Female	42.2% (140,035/331694)	55.5% (47,546/85652)	< 0.001	44.9% (187,581/417346)
	Male	57.8% (191,659/331694)	44.5% (38,106/85652)		55.1% (229,765/417346)
Income level	< 30%	28.5% (94,614/331694)	30% (25,732/85652)	< 0.001	28.8% (120,346/417346)
	30–80%	34.9% (115,713/331694)	40.5% (34,656/85652)		36% (150,369/417346)
	> 80%	36.6% (121,367/331694)	29.5% (25,264/85652)		35.1% (146,631/417346)
**Physical examination**					
Body habitus	Normal (BMI < 25 kg/m^2^)	66.2% (219,447/331529)	64.2% (54,942/85617)		65.8% (274,419/417217)
	Overweight (25–30 kg/m^2^)	31.3% (103,922/331529)	32.6% (27,933/85617)		31.6% (131,855/417217)
	Obese (> 30 kg/m^2^)	2.5% (8130/331529)	3.2% (2742/85617)		2.6% (10,943/417217)
Systolic BP (mmHg)		126.59 (17.19)	126.54 (17.17)	0.33	126.58 (17.18)
Diastolic BP (mmHg)		79.18 (11.15)	78.37 (10.82)	< 0.001	79.02 (11.09)
**Blood test**					
AST (IU/L)		26.55 (15.92)	26.47 (17.37)	0.25	26.53 (16.23)
ALT (IU/L)		25.52 (19.45)	24.9 (20.63)	< 0.001	25.39 (19.7)
GGT (IU/L)		37.7 (51.85)	36.58 (53.85)	< 0.001	37.47 (52.27)
Total cholesterol (mg/dL)		198.32 (36.84)	199.45 (37.84)	< 0.001	198.55 (37.05)
Fasting blood glucose (mg/dL)		97.87 (28.9)	99.53 (28.12)	< 0.001	98.21 (28.75)
Hemoglobin (g/dL)		13.94 (1.49)	13.65 (1.51)	< 0.001	13.89 (1.5)
**Habit**					
Smoking (pack-year)		5.96 (11.53)	4.6 (11.11)	< 0.001	5.68 (11.46)
Alcohol consumption (mL/week)		8.7 (18.7)	7.7 (19.1)	< 0.001	8.5 (18.8)
Exercise	Rarely	50.1% (162,668/324506)	56.4% (46,716/82888)	< 0.001	51.4% (209,384/407394)
	1–2 per week	26.9% (87,443/324506)	22.2% (18,437/82888)		26% (105,880/407394)
	3–4 per week	12.1% (39,341/324506)	10.4% (8647/82888)		11.8% (47,988/407394)
	5–6 per week	3.2% (10,384/324506)	3.1% (2587/82888)		3.2% (12,971/407394)
	Almost everyday	7.6% (24,670/324506)	7.8% (6501/82888)		7.7% (31,171/407394)
**Family history**					
Liver disease		2.81% (8563/305086)	2.64% (2040/77356)	0.01	2.77% (10,603/382442)
Hypertension		9.16% (28,072/306539)	9.69% (7548/77870)	< 0.001	9.27% (35,620/384409)
Stroke		5.47% (16,738/305817)	5.55% (4310/77600)	0.38	5.49% (21,048/383417)
Heart disease		2.39% (7278/305106)	2.57% (1992/77365)	< 0.001	2.42% (9270/382471)
Diabetes mellitus		6.45% (19,729/306048)	6.8% (5280/77653)	< 0.001	6.52% (25,009/383701)
Cancer		13.14% (40,389/307443)	13.45% (10,498/78065)	0.02	13.2% (50,887/385508)
**Underlying medical condition**^**a**^					
Diabetes mellitus		6.07% (20,139/331694)	8.83% (7565/85652)	< 0.001	6.64% (27,704/417346)
Dyslipidemia		5.95% (19,725/331694)	10.45% (8950/85652)	< 0.001	6.87% (28,675/417346)
Chronic hepatitis virus infection		2.57% (8530/331694)	3.71% (3176/85652)	< 0.001	2.8% (11,706/417346)
Human Immunodeficiency virus		6.73% (22,314/331694)	10.6% (9079/85652)	< 0.001	7.52% (31,393/417346)
Schizophrenic or delusional disorders, or mental disorders due to psychoactive substance use		15.59% (51,714/331694)	25.57% (21,905/85652)	< 0.001	17.64% (73,619/417346)
Chronic liver disease		5.4% (17,927/331694)	8.21% (7033/85652)	< 0.001	5.98% (24,960/417346)
Alcoholic fatty liver disease		1.95% (6480/331694)	2.75% (2353/85652)	< 0.001	2.12% (8833/417346)
Non-alcoholic fatty liver diseases		3.01% (9996/331694)	5.07% (4345/85652)	< 0.001	3.44% (14,341/417346)

For continuous variables, numbers in each cell and parentheses are mean and standard deviation, respectively. *BMI* body mass index, *BP* blood pressure, *AST* aspartate aminotransferase, *ALT* alanine aminotransferase, *GGT* gamma-glutamyl transferase

^a^This is not a complete list. The full list can be found in Supplementary Table 1

#### Risk factors

Including irrelevant input variables in a machine learning model likely results in overfitting and can undermines the generalizability of a prediction model [19]. Thus, variable selection was performed using Cox proportional hazard (CoxPH) regression in the training cohort. First, multicollinearity among the variables was examined by calculating variance inflation factors (VIFs). Systolic/diastolic blood pressure and aspartate transaminase (AST)/alanine transaminase (ALT) were determined to have strong correlation as they showed VIFs > 2.5 (Supplementary Table 3). Thus, mean average was calculated and used instead of systolic or diastolic blood pressure, and AST was discarded as ALT is more specific to liver disease. Next, using the variables that showed statistically significant (*p* < 0.05) associations with HCC development in the univariable analysis as input variables, the multivariable analysis was performed to identify independent predictors. In order to select stable predictors, this selection process was repeated 1000 times with different datasets resampled by bootstrapping the training dataset, and only variables that were chosen as independent predictors for HCC in > 85% of the 1000 datasets were selected as the final predictors.

#### Hazard ratio of predictors for HCC

In the multivariable CoxPH regression, the hazard ratios (HRs) of the selected predictors were estimated with and without other cancers included as the competing risk. Subdistribution hazard with the competing risk was estimated using the methodology by Fine and Gray [20].

#### Training machine learning models in the training cohort

Random survival forest (RSF) algorithm was used for predicting the probability of and the time to HCC occurrence, with non-HCC cancers included as competing risks [21]. In addition, we tested whether an ensemble of RSF and multivariate extreme gradient boosting (XGBoost) algorithm could improve the accuracy of probability prediction. Hyperparameters were optimized using grid search by assessing out-of-bag errors for RSF and by 10-fold cross validation with area under receiver operating characteristics curve (AUC) as an evaluation metric for XGBoost. Optimal hyperparameters found were ntree = 120, mtry = 1, and nodesize = 6 for RSF, and max.depth = 5, eta = 0.1, min_child_weight = 1, gamma = 0, lambda = 0, and nrounds = 108 for XGBoost, with other parameters set to default. With the selected predictors and the optimal hyperparameters, the models were fit to the training dataset. In prediction of the probability of the development of HCC, the performances of RSF, XGBoost, and both were compared in terms of Brier skill score, AUC, and calibration plot, and the best model was chosen. Although the Brier score is a proper score function that measures the accuracy of probabilistic predictions, it does not tell us how accurate the predictions are compared with anything else, which may result in misleading results especially when a target outcome is rare as in this study. Thus, we used Brier skill score that assess the accuracy of predictions compared to a reference prediction of always predicting ‘no HCC development’: *Brier skill score* = 1 − (*Brier score*/*Reference Brier Score*).

#### Validation in the test cohort

The performance of the final model was evaluated in the test cohort: AUC, Brier skill score, and calibration plot for the probability, and concordance index (*C*-index) for the time to HCC development. The sensitivity, specificity, and accuracy for HCC development were calculated at the optimal cutoff probability obtained from AUC analysis. Kaplan-Meier curve with log-rank test was used to compare the survival curves between three groups divided according to the predicted probability: low-risk (< 5%), intermediate-risk (5–20%), and high-risk (> 20%) groups.

#### Prediction models in subgroups

Using the same methods explained above, we also developed and validated models with subgroups of patients with DM, alcoholic fatty liver disease (AFLD), and non-alcoholic fatty liver disease (NAFLD).

## Results

### Study population

The final study population consisted of 417,346 examinees, with 331,694 (79.5%) in the training cohort and 85,652 (20.5%) in the test cohort (Fig. 1). The age ranged from 42 to 82 (mean, 55) years at the time of the examination, and the ratio of males to females was 5.5:4.5. Most of the variables were different in frequency or mean between the training and test cohorts (Table 1 and Supplementary Table 1). The median follow-up time was 11.1 years (up to 12.0 years) in the training cohort and 9.1 years (up to 10.0 years) in the test cohort. Of the total examinees, 0.5% (1799/331,694 in the training cohort and 390/85,652 in the test cohort) were diagnosed with HCC, and 8.4% (27,856/331,694) and 7.9% (6732/85,652) in the training cohort and the test cohort were diagnosed with other cancers during the follow-up period, respectively.

### Selected predictors and their hazard ratios for HCC

Stable predictors that showed significant associations with the risk of HCC development in > 85% of 1000 different resampled datasets were age, sex, obesity, income level, the family history of chronic liver disease, ALT, gamma-glutamyl transpeptidase (GGT), total blood cholesterol level, and preexisting chronic liver disease, chronic hepatitis virus infection, HIV infection, DM, dyslipidemia, or schizophrenic/delusional disorders or mental disorders due to psychoactive substance use (Supplementary Table 4).

**Fig. 2:**
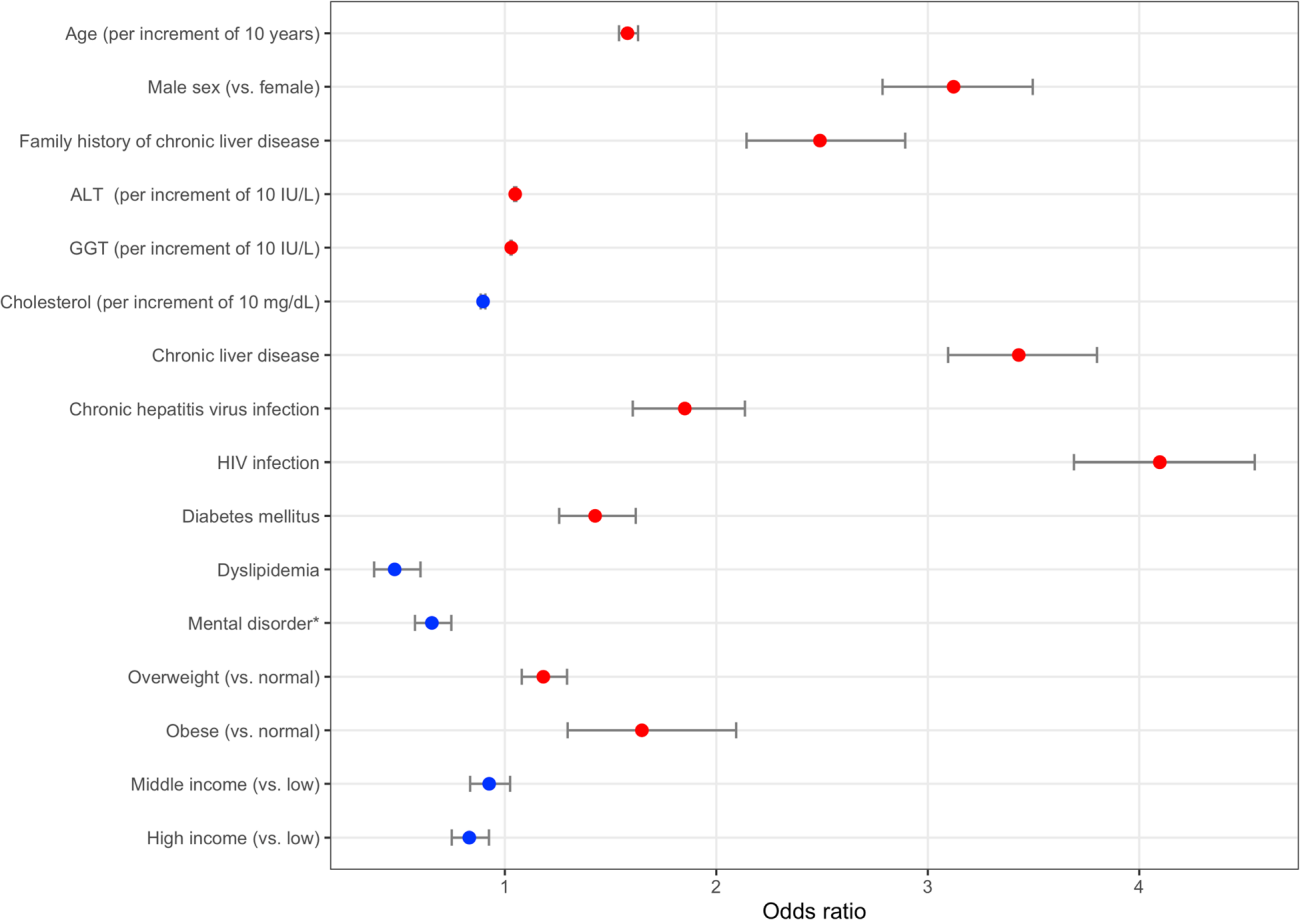
Forest plot of odds ratios. The odds ratios of the final predictors for HCC development from the multivariable Cox proportional hazard regression in the training cohort are presented as red (associated with increased risk) or blue (associated with decreased risk) dots. The horizontal error bars indicate 95% confidence intervals. ^*^mental disorder includes schizophrenic or delusional disorders, or mental disorders due to psychoactive substance use

In the multivariable CoxPH regression, older age (HR, 1.581; per increment of 10 years), male sex (HR, 3.122), family history of chronic liver disease (HR, 2.490), obesity (HR, 1.648), higher levels of ALT (HR, 1.049; per increment of 10 IU/L) or GGT (HR, 1.030; per increment of 10 IU/L), and preexisting chronic liver disease (HR, 3.430), chronic hepatitis virus infection (HR, 1.851), HIV infection (HR, 4.097), and DM (HR, 1.427) were associated with increased risk, whereas a higher level of total cholesterol (HR, 0.897; per increment of 10 mg/dL), high income level (HR, 0.832), and preexisting dyslipidemia (HR, 0.479) or schizophrenic/delusional disorders or mental disorders due to psychoactive substance use (HR, 0.655) were associated with decreased risk of HCC development (*p* < 0.001 for all variables). HRs were not significantly affected by whether or not the development of non-HCC cancers was considered competing risks (Table 2 and Fig. 2).

**Table 2:** Multivariable Cox proportional hazard regression for HCC with and without other cancers included as competing risks in the training cohort

	No competing risk			Competing risk included		
	HR	95% CI	*p*-value	HR	95% CI	*p*-value
Age^a^	1.581	1.540–1.629	< 0.001	1.542	1.493–1.581	< 0.001
Male sex (vs. female)	3.122	2.786–3.496	< 0.001	3.040	2.710–3.401	< 0.001
Family history of chronic liver disease	2.490	2.143–2.893	< 0.001	2.487	2.099–2.948	< 0.001
ALT^a^	1.049	1.044–1.054	< 0.001	1.048	1.036–1.061	< 0.001
GGT^a^	1.030	1.027–1.032	< 0.001	1.029	1.026–1.032	< 0.001
Total cholesterol^a^	0.897	0.886–0.908	< 0.001	0.898	0.887–0.910	< 0.001
Chronic liver disease	3.430	3.096–3.800	< 0.001	3.470	3.116–3.863	< 0.001
Chronic hepatitis virus infection	1.851	1.605–2.135	< 0.001	1.862	1.567–2.213	< 0.001
HIV infection	4.097	3.691–4.546	< 0.001	4.057	3.640–4.522	< 0.001
Diabetes mellitus	1.427	1.257–1.619	< 0.001	1.430	1.255–1.629	< 0.001
Dyslipidemia	0.479	0.382–0.601	< 0.001	0.458	0.359–0.585	< 0.001
Schizophrenic or delusional disorders, or mental disorders due to psychoactive substance use	0.655	0.575–0.747	< 0.001	0.645	0.563–0.738	< 0.001
Overweight (vs. normal)	1.182	1.080–1.294	< 0.001	1.190	1.084–1.308	< 0.001
Obese (vs. normal)	1.648	1.297–2.094	< 0.001	1.662	1.304–2.118	< 0.001
Middle income (vs. low)	0.926	0.836–1.025	0.138	0.925	0.831–1.030	0.150
High income (vs. low)	0.832	0.749–0.925	< 0.001	0.834	0.747–0.931	0.001

*HCC* hepatocellular carcinoma, *HR* hazard ratio, *CI* confidence interval, *ALT* alanine aminotransferase, *GGT* gamma-glutamyl transferase, *HIV* human immunodeficiency

^a^per increment of 10 years, IU/L, or mg/dL

### Machine learning

#### Probability prediction

**Fig. 3:**
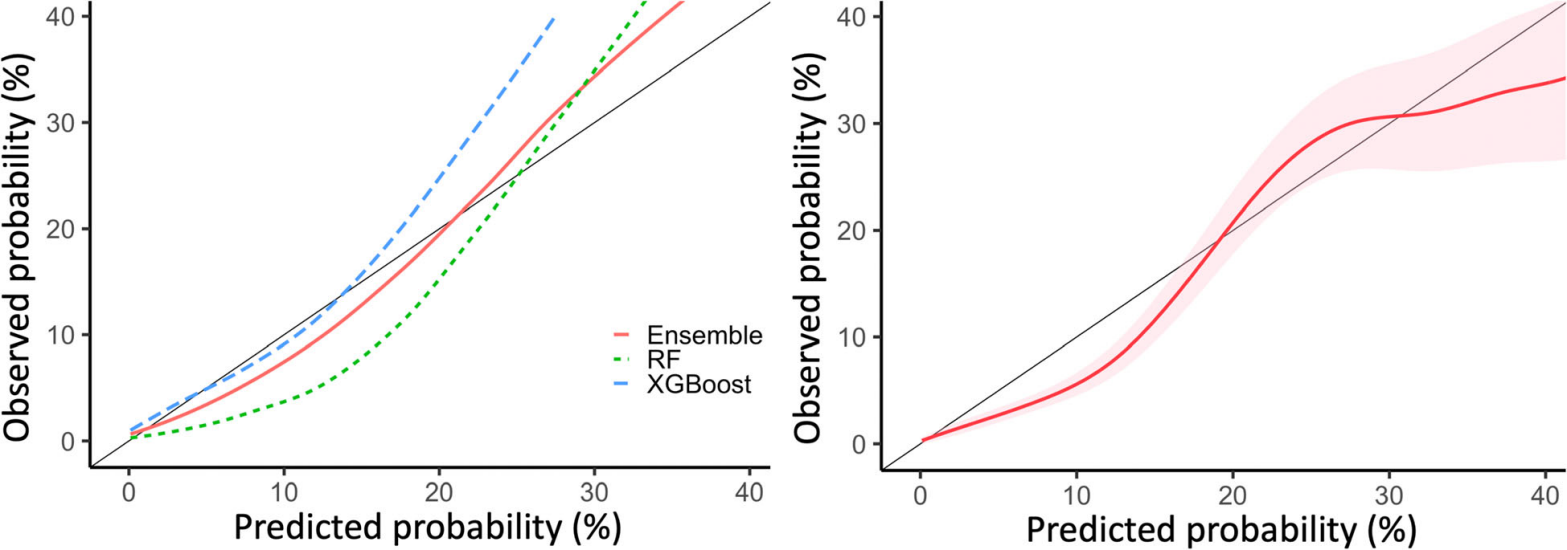
Calibration curves. The left panel shows calibration curves in the training cohort (Black, ideal line; Blue, extreme gradient boosting (XGBoost); Green, Random Forest (RF); Red: Ensemble model). An ensemble of XGBoost and RF showed the best calibration curve. Thus, the ensemble model was chosen as our final model. The right panel shows the calibration curve of the final model in the test cohort, with the area of pinkish shades indicating 95% confidence interval

In the training cohort, the XGBoost showed better performance than the RSF model in predicting the risk of HCC development. For discriminating whether HCC will develop or not, the AUCs (±standard deviation) of the XGBoost and RSF models were 0.882 (±0.013) and 0.871 (±0.019) in the cross validation and out-of-bag validation, respectively. In terms of calibration, the Brier skill scores were 0.109 and 0.062, which can be interpreted as 10.9 and 6.2% improvement in Brier score compared to the baseline model, respectively. An ensemble of XGBoost and RSF showed slightly better AUC (0.892 [±0.011]) and Brier skill score (0.112) to XGBoost alone, and it was determined to show the best calibration curve (Fig. 3). Therefore, the ensemble model was chosen as our final model (Table 3).

**Table 3:** Performances of machine learning models in prediction of the probability of and the time to the development of HCC

Model	Evaluation metric	CV or OOB error in the training cohort	Validation in the test cohort
		Value (±SD)	AUC (95% CI)
Probability of developing HCC within 10 years			
Random survival forest	AUC	0.871 (±0.019)	
	BSS	0.062	
Extreme gradient boosting	AUC	0.882 (±0.013)	
	BSS	0.109	
Ensemble of two models	AUC	0.892 (±0.011)	0.873 (0.860–0.885)
	BSS	0.112	0.078
Time to cancer occurrence if HCC develops			
Cox proportional hazard	C-index	0.843 (±0.006)	0.828 (0.819–0.838)
Random survival forest	C-index	0.881 (±0.010)	0.857 (0.850–0.864)

*HCC* hepatocellular carcinoma, *CV* cross validation, *OOB* out-of-bag, *SD* standard deviation, *AUC* area under receiver operating characteristics curve, *CI* confidence interval, *BSS* Brier skill score, *C-index* concordance index

**Fig. 4:**
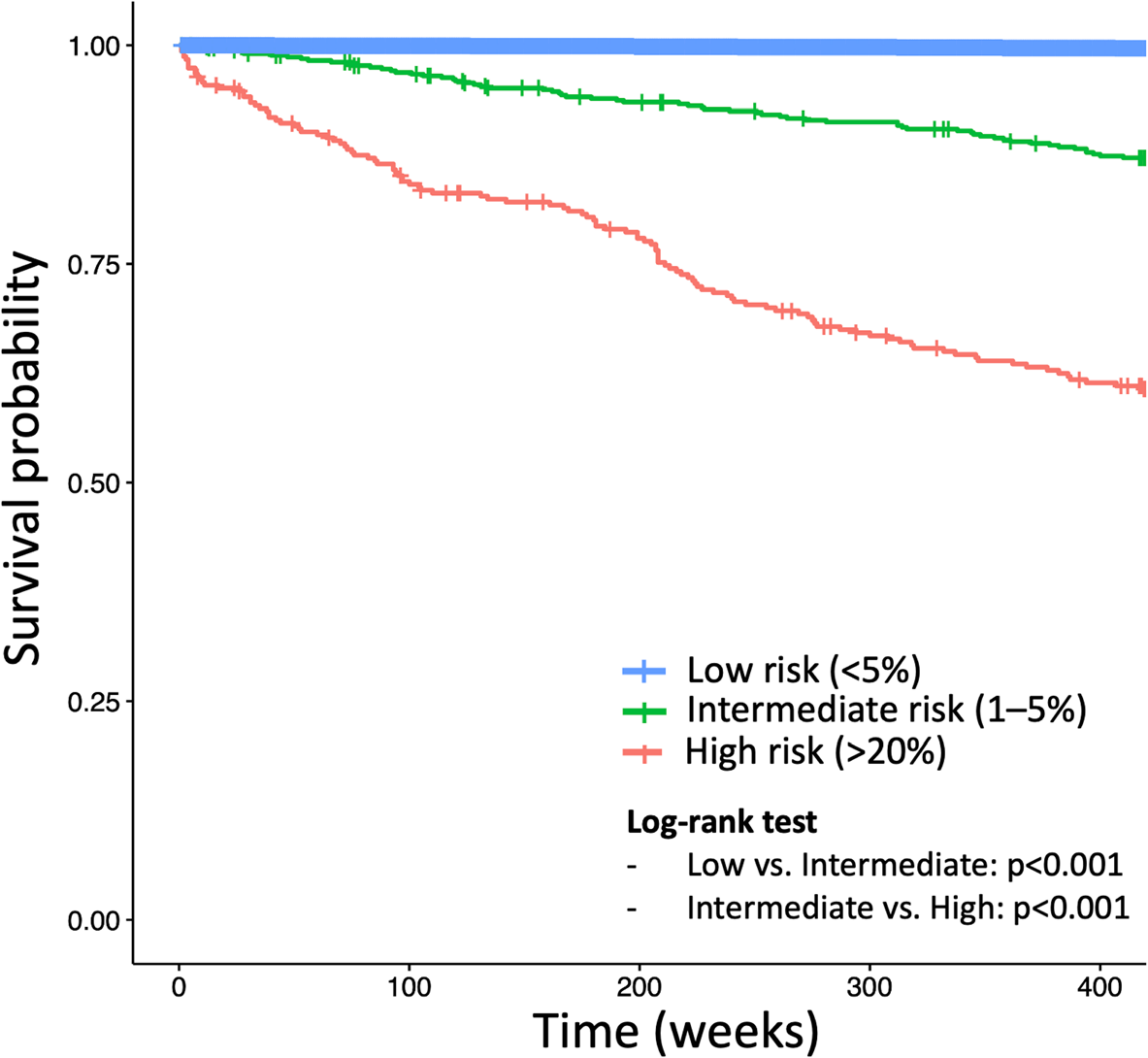
Survival curves of the three groups according to the risk of developing HCC predicted on our model in the test cohort. The time to HCC development were significantly different between the groups when the test cohort was divided into three groups based on their predicted probability of developing HCC within 9 years: low-risk, probability of < 5%; intermediate-risk, 5–20%; and high-risk, > 20%

In the test cohort, our prediction model showed good calibration with a trend of mild underestimation with probabilities < 20% (Fig. 3). The AUC was 0.873 (95% CI, 0.860–0.885). The Brier skill score was 0.078. Using 1% as a cutoff probability, the sensitivity, specificity, and accuracy were 71.8% (95% CI, 71.4–72.2), 88.4% (95% CI, 88.1–88.7), and 88.4% (95% CI, 88.2–88.6), respectively. In the Kaplan-Meier curve with log-rank test, the curves for the three risk groups (i.e., low, < 5%; intermediate, 5–20%; and high, > 20%) were separated well (*p* < 0.001 for all comparisons) in the test cohort (Fig. 4).

#### Time-to-event prediction

**Fig. 5:**
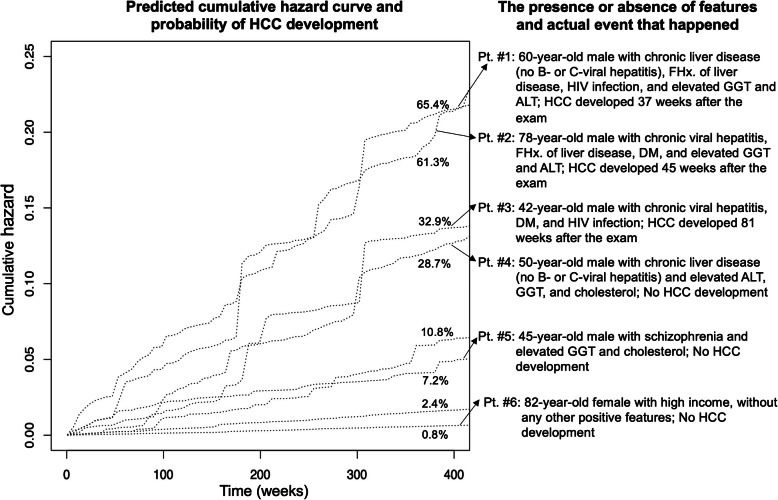
Representative cases with different predicted risks. In the left panel, cumulative hazard curves of eight screening examinees are shown with the predicted risks of developing hepatocellular carcinoma. In the right panel, the risk factors they had and the actual events that happened to them are summarized. FHx = family history, HIV = human immunodeficiency virus, GGT = gamma-glutamyl transferase, ALT = alanine aminotransferase, HCC = hepatocellular carcinoma, DM = diabetes mellitus

The median time to HCC development was 294 weeks (5.6 years) in the training cohort and 235 weeks (4.5 years). In prediction of the time to HCC development, the RSF model showed better discriminative ability than CoxPH in the test cohort with the c-indices of CoxPH and RSF being 0.828 (95% CI, 0.819–0.838) and 0.857 (95% CI, 0.850–0.864), respectively. Representative cases of individual predictions of the time-to-HCC by RSF are shown in Fig. 5.

#### Performances of the models developed in subgroups

In the subgroups of patients with DM, NAFLD, and AFLD, the prediction models showed slightly decreased but comparable performances in predicting the probability of HCC occurrence. In the validation using the test cohort, the AUC was 0.851 (95% CI, 0.794–0.863), 0.853 (95% CI, 0.801–0.822), 0.849 (95% CI, 0.837–0.861) in patients with DM, NAFLD, and AFLD, respectively (Table 4).

**Table 4:** Performance of ensemble machine learning model for probability of HCC development within 9 years in subgroups of patients with preexisting medical conditions: alcoholic or non-alcoholic fatty liver disease, and diabetes mellitus

Model	Subset of selected risk factors	CV or OOB error in the training cohort	Validation in the test cohort
		Value (±SD)	AUC (95% CI)
DM subgroup	Age, sex, ALT, GGT, CLD, HIV, Schizophrenia	0.873 (±0.006)	0.851 (0.794–0.863)
NAFLD subgroup	Age, Sex, Income, ALT, GGT, cholesterol, CLD, CVH, HIV	0.882 (±0.006)	0.853 (0.801–0.822)
AFLD subgroup	Age, Sex, FHx of CLD, ALT, cholesterol, CLD, CVH, HIV	0.874 (±0.006)	0.849 (0.837–0.861)

*HCC* hepatocellular carcinoma, *CV* cross validation, *OOB* out-of-bag, *SD* standard deviation, *AUC* area under receiver operating characteristics curve, *CI* confidence interval, *DM* diabetes mellitus, *NAFLD* non-alcoholic fatty liver disease, *AFL* alcoholic fatty liver disease, *ALT* alanine aminotransferase, *GGT* gamma-glutamyl transferase, *CLD* chronic liver disease, *HIV* human immunodeficiency virus, *CVH* chronic viral hepatitis, *FHx* family history

## Discussion

In this study, we developed a machine learning model to predict the risk of developing HCC within the following 9 years in an individual health screening examinee, based on the information available from the examination results and the history of medical service use. The model showed good calibration and discrimination in the test. Furthermore, the models developed in the subgroups of patients with DM, AFLD, or NAFLD showed similarly good performances.

The previously published models to predict the risk of HCC development were mostly for patients with chronic liver disease, which are well-summarized in other reviews [11, 12]. Of the models published so far, to the best of our knowledge, only three were developed on general populations. Michikawa et al. used age, sex, alcohol or coffee consumption, obesity, and the presence of DM, or HBV or HCV infection as independent predictors for their prediction model developed in a cohort of 17,654 Japanese undergoing health examination [22]. Wen et al. developed a model to predict the risk of HCC development based on age, sex, alcohol consumption, ALT, AST, and alpha-feto protein, and the presence of DM, or HBV or HCV infection using a cohort of 428,584 health screening examinee in Taiwan [23]. A recent study conducted in Korea used the same cohort as ours to develop a prediction model with age, sex, smoking, DM, total cholesterol, and ALT used as predictors; however, this model can only be used for people without traditional risk factors (i.e., chronic viral hepatitis and liver cirrhosis) [24]. All the previous studies used CoxPH regression, while we used the machine learning algorithms (i.e., RSF and XGBoost) and found that they may be superior to the conventional CoxPH in the risk prediction. In addition, all the previous three models simply included known risk factors as potential predictors during model development, while we made efforts to extract important information from the insurance claim data that otherwise would have been discarded. As a result, income level, schizophrenic or delusional disorders, and HIV infection—factors that had not been used by the previous models—were included as important predictors in our model.

However, we were cautious in identifying potential predictors from the data. Complex machine learning algorithms can be so flexible that they pick up meaningless or noisy signals from input data to make good predictions only in a certain dataset but fail to generalize to other datasets with different noises. Therefore, by the rigorous variable selection process, we aimed to remove noisy signals, that is, non-significant, unstable input variables; in our results many seemingly irrelevant underlying diseases such as hemorrhoid or chronic rhinitis were frequently selected as independent risk factors for HCC in resampled datasets (Supplementary Table 4).

Older age, male sex, chronic liver disease, heavy alcohol consumption, diabetes, obesity, and HIV infection are well-known risk factors for HCC [25, 26]. All of these risk factors were independent predictors in our cohort as well. An exception was drinking habit by questionnaire, which was not selected as a final predictor in our model, which is consistent with the result of the previous study that used the same cohort for model development [24]. The non-significance of alcohol consumption in the presence of other strong predictors may be attributed partly to the unreliability of examinees’ answers to the questionnaire used in a health screening examination, as a previous study pointed out [27]. Although family history of liver cancer is also a known risk factor for HCC [28, 29], it was not selected as a predictor in our model. The presence or absence of family history of cancer was also asked in our health screening questionnaire, but it includes all types of cancer, which is probably the reason that it was not included as a significant risk factor.

In contrast to DM, underlying dyslipidemia and higher total cholesterol were associated with the lower risk in our cohort. This opposite associations between DM, dyslipidemia, and HCC are in line with the results of an epidemiologic study of HCC and metabolic risk factors in a nationwide Taiwan cohort [30]. This may be partly explained by that in this study dyslipidemia was diagnosed when both the diagnosis and the use of lipid-lowering drugs were confirmed (Supplementary Table 2), and current evidence suggests that statin use could contribute to a decline in HCC incidence [25, 31]. However, hypercholesterolemia without taking lipid-lowering drugs was also an independent risk factor [30]. More research is warranted on the effect and mechanism of dyslipidemia on the risk of HCC development and prognosis.

Interpretation of the lower risk of HCC in patients with mental disorders due to psychoactive substance use or schizophrenic and delusional disorders is hampered by the fact that those diagnoses were considered sensitive personal information and grouped together under the unidentified code in our dataset. However, as mental disorders due to use of alcohol, which is most commonly used psychoactive substance, probably affected the outcome towards an increased risk, schizophrenic and delusional disorders were likely attributed to the decreased risk of HCC. Especially, schizophrenia has been reported by a meta-analysis study to be protective against HCC development [32]. Some investigators suggested the correlation between tumor suppressor genes and schizophrenia as possible explanation of its potential protective effect against cancer [33].

A major limitation of our prediction model is that it was developed and validated using a single ethnic (i.e., Asian) population from a single country, without an independent external validation. Thus, the generalizability of the model to other countries or ethnic groups is not guaranteed. However, we believe that our approach (i.e., machine learning predictor based on the claim and health screening data) can be applied to various cohorts similarly and used to produce their own, even multi-national, prediction models. In addition, we could not include subjects from recent years, since this study required years of follow-up by its design, and the NHIS-HEALS data only contain information until 2015. This may have led to the biased model that does not fully reflect the current trend in the prevalence and characteristics of some risk factors such as obesity, alcohol consumption, and fatty liver disease. Lastly, some diagnoses were masked and grouped together for the protection of sensitive personal information. We expect that more detailed information from the national health insurance database will be made available for research purposes in the future.

## Conclusions

In conclusion, machine learning could be used to develop a prediction model for the risk of HCC development in individual health screening examinees, based on the information retrieved from the examination results and healthcare claim data.

## Supplementary Information

**Additional file 1.**

## Data Availability

The datasets generated and/or analysed during the current study are not publicly available due to the provisions of the National Health Insurance Service (NHIS), but other materials are available from the corresponding author on reasonable request.

## Abbreviations

HCC: Hepatocellular carcinoma
NHIS-HEALS: National health insurance service-national health screening
HIV: Human immunodeficiency viruse
CoxPH: Cox proportional hazard
AST: Aspartate transaminase
ALT: Alanine transaminase
HR: Hazard ratio
RSF: Random survival forest
XGBoost: Extreme gradient boosting
AUC: Area under receiver operating characteristics curve
DM: Diabetes mellitus
AFLD: Alcoholic fatty liver disease
NAFLD: Non-alcoholic fatty liver disease
GGT: Gamma-glutamyl transpeptidase.
