# Supplementary material for: Prediction of the risk of developing hepatocellular carcinoma in health screening examinees: a Korean cohort study
Source: BMC Cancer. 2021 Jun 29;21:755. doi: 10.1186/s12885-021-08498-w (PMC8243543; doi:10.1186/s12885-021-08498-w)
Supplement: Supplementary file 1 — Additional file 1. [file 12885_2021_8498_MOESM1_ESM.docx]

Supplementary materials

**Supplementary Table 1.** A full list of baseline characteristics

| Variable | | Training cohort (n=331694) | Test cohort (n=85652) | *p*-value | Total (n=417346) |
| --- | --- | --- | --- | --- | --- |
| **Sociodemographic characteristics** | | | | | |
| Age (years) | | 54.3 (9.28) | 57.7 (9.6) | <0.001 | 55.0 (9.45) |
| Sex | Female | 42.2% (140035/331694) | 55.5% (47546/85652) | <0.001 | 44.9% (187581/417346) |
|  | Male | 57.8% (191659/331694) | 44.5% (38106/85652) |  | 55.1% (229765/417346) |
| Residence | Metropolitan | 16.6% (55179/331694) | 17% (14522/85652) | <0.001 | 16.7% (69701/417346) |
|  | Urban or suburban | 28.2% (93582/331694) | 25% (21389/85652) |  | 27.5% (114971/417346) |
|  | Rural | 55.2% (182933/331694) | 58.1% (49741/85652) |  | 55.8% (232674/417346) |
| Income level | <30% | 28.5% (94614/331694) | 30% (25732/85652) | <0.001 | 28.8% (120346/417346) |
|  | 30-80% | 34.9% (115713/331694) | 40.5% (34656/85652) |  | 36% (150369/417346) |
|  | >80% | 36.6% (121367/331694) | 29.5% (25264/85652) |  | 35.1% (146631/417346) |
| **Physical examination** | | | | | |
| Body habitus | Normal (BMI< 25 kg/m^2^) | 66.2% (219447/331529) | 64.2% (54942/85617) |  | 65.8% (274419/417217) |
|  | Overweight (25-30 kg/m^2^) | 31.3% (103922/331529) | 32.6% (27933/85617) |  | 31.6% (131855/417217) |
|  | Obese (> 30 kg/m^2^) | 2.5% (8130/331529) | 3.2% (2742/85617) |  | 2.6% (10943/417217) |
| Systolic BP (mmHg) | | 126.59 (17.19) | 126.54 (17.17) | 0.33 | 126.58 (17.18) |
| Diastolic BP (mmHg) | | 79.18 (11.15) | 78.37 (10.82) | <0.001 | 79.02 (11.09) |
| **Blood test** | | | | | |
| AST (IU/L) | | 26.55 (15.92) | 26.47 (17.37) | 0.25 | 26.53 (16.23) |
| ALT (IU/L) | | 25.52 (19.45) | 24.9 (20.63) | <0.001 | 25.39 (19.7) |
| GGT (IU/L) | | 37.7 (51.85) | 36.58 (53.85) | <0.001 | 37.47 (52.27) |
| Total cholesterol (mg/dL) | | 198.32 (36.84) | 199.45 (37.84) | <0.001 | 198.55 (37.05) |
| Fasting blood glucose (mg/dL) | | 97.87 (28.9) | 99.53 (28.12) | <0.001 | 98.21 (28.75) |
| Hemoglobin (g/dL) | | 13.94 (1.49) | 13.65 (1.51) | <0.001 | 13.89 (1.5) |
| **Urine stick test** | | | | | |
| Urine pH level (pH) | | 6.09 (0.64) | 6.09 (0.56) | 0.99 | 6.09 (0.62) |
| Urine Glucose | Negative or weak | 97.4% (321935/330570) | 97.4% (82994/85176) | 0.59 | 97.4% (404929/415746) |
|  | Medium | 0.9% (3137/330570) | 0.9% (808/85176) |  | 0.9% (3945/415746) |
|  | Strong | 1.7% (5498/330570) | 1.6% (1374/85176) |  | 1.7% (6872/415746) |
| Urine Occult blood | Negative or weak | 93.1% (307889/330554) | 90.1% (76772/85172) | <0.001 | 92.5% (384661/415726) |
|  | Medium | 3.8% (12614/330554) | 5.2% (4434/85172) |  | 4.1% (17048/415726) |
|  | Strong | 3% (10051/330554) | 4.7% (3966/85172) |  | 3.4% (14017/415726) |
| Urine protein | Negative or weak | 98.2% (324482/330579) | 97.8% (83315/85175) | <0.001 | 98.1% (407797/415754) |
|  | Medium | 1.2% (4122/330579) | 1.4% (1228/85175) |  | 1.3% (5350/415754) |
|  | Strong | 0.6% (1975/3305/79) | 0.7% (632/85175) |  | 0.6% (2607/415754) |
| **Habit** | | | | | |
| Smoking (pack-year) | | 5.96 (11.53) | 4.6 (11.11) | <0.001 | 5.68 (11.46) |
| Alcohol consumption (mL/week) | | 8.7 (18.7) | 7.7 (19.1) | <0.001 | 8.5 (18.8) |
| Exercise | Rarely | 50.1% (162668/324506) | 56.4% (46716/82888) | <0.001 | 51.4% (209384/407394) |
|  | 1-2 per week | 26.9% (87443/324506) | 22.2% (18437/82888) |  | 26% (105880/407394) |
|  | 3-4 per week | 12.1% (39341/324506) | 10.4% (8647/82888) |  | 11.8% (47988/407394) |
|  | 5-6 per week | 3.2% (10384/324506) | 3.1% (2587/82888) |  | 3.2% (12971/407394) |
|  | Almost everyday | 7.6% (24670/324506) | 7.8% (6501/82888) |  | 7.7% (31171/407394) |
| **Family history** | | | | | |
| Liver disease | | 2.81% (8563/305086) | 2.64% (2040/77356) | 0.01 | 2.77% (10603/382442) |
| Hypertension | | 9.16% (28072/306539) | 9.69% (7548/77870) | <0.001 | 9.27% (35620/384409) |
| Stroke | | 5.47% (16738/305817) | 5.55% (4310/77600) | 0.38 | 5.49% (21048/383417) |
| Heart disease | | 2.39% (7278/305106) | 2.57% (1992/77365) | <0.001 | 2.42% (9270/382471) |
| Diabetes mellitus | | 6.45% (19729/306048) | 6.8% (5280/77653) | <0.001 | 6.52% (25009/383701) |
| Cancer | | 13.14% (40389/307443) | 13.45% (10498/78065) | 0.02 | 13.2% (50887/385508) |
| **Underlying medical condition** | | | | | |
| Diabetes mellitus | | 6.07% (20139/331694) | 8.83% (7565/85652) | <0.001 | 6.64% (27704/417346) |
| Dyslipidemia | | 5.95% (19725/331694) | 10.45% (8950/85652) | <0.001 | 6.87% (28675/417346) |
| Hypertension | | 19.15% (63511/331694) | 28.11% (24073/85652) | <0.001 | 20.99% (87584/417346) |
| Chronic hepatitis virus infection | | 2.57% (8530/331694) | 3.71% (3176/85652) | <0.001 | 2.8% (11706/417346) |
| Human Immunodeficiency virus | | 6.73% (22314/331694) | 10.6% (9079/85652) | <0.001 | 7.52% (31393/417346) |
| Schizophrenic or delusional disorders, or mental disorders due to psychoactive substance use | | 15.59% (51714/331694) | 25.57% (21905/85652) | <0.001 | 17.64% (73619/417346) |
| Chronic liver disease | | 5.4% (17927/331694) | 8.21% (7033/85652) | <0.001 | 5.98% (24960/417346) |
| Stroke | | 0.02% (72/331694) | 0.08% (67/85652) | <0.001 | 0.03% (139/417346) |
| Ischemic heart disease | | 2.67% (8859/331694) | 4.72% (4046/85652) | <0.001 | 3.09% (12905/417346) |
| Atrial fibrillation | | 0.41% (1351/331694) | 0.69% (595/85652) | <0.001 | 0.47% (1946/417346) |
| Gastrointestinal bacterial infection | | 11.59% (38452/331694) | 19.17% (16422/85652) | <0.001 | 13.15% (54874/417346) |
| Other bacterial infection | | 4.06% (13474/331694) | 6.51% (5577/85652) | <0.001 | 4.56% (19051/417346) |
| Other viral infection | | 3.57% (11835/331694) | 5.08% (4354/85652) | <0.001 | 3.88% (16189/417346) |
| Fungal infection | | 21.21% (70368/331694) | 24.1% (20651/85652) | <0.001 | 21.8% (91019/417346) |
| Gastrointestinal benign tumors | | 1.5% (4979/331694) | 3.16% (2703/85652) | <0.001 | 1.84% (7682/417346) |
| Thyroid benign tumors | | 0.42% (1379/331694) | 0.9% (768/85652) | <0.001 | 0.51% (2147/417346) |
| Other benign tumors | | 3.11% (10323/331694) | 5.66% (4847/85652) | <0.001 | 3.63% (15170/417346) |
| Blood disorders | | 3.82% (12674/331694) | 7.1% (6081/85652) | <0.001 | 4.49% (18755/417346) |
| Thyroid diseases | | 4.2% (13928/331694) | 7.8% (6686/85652) | <0.001 | 4.9% (20614/417346) |
| Other endocrine diseases | | 0.41% (1357/331694) | 0.77% (661/85652) | <0.001 | 0.48% (2018/417346) |
| Other metabolic diseases | | 1.01% (3341/331694) | 2.07% (1775/85652) | <0.001 | 1.23% (5116/417346) |
| Mood disorders | | 4.48% (14854/331694) | 7.72% (6613/85652) | <0.001 | 5.14% (21467/417346) |
| Mental disorder due to organic causes | | 0.23% (752/331694) | 0.64% (550/85652) | <0.001 | 0.31% (1302/417346) |
| Headache | | 10.89% (36130/331694) | 18.79% (16091/85652) | <0.001 | 12.51% (52221/417346) |
| Sleep disorders | | 2.39% (7926/331694) | 4.88% (4184/85652) | <0.001 | 2.9% (12110/417346) |
| Neuropathy | | 8.58% (28464/331694) | 16.23% (13905/85652) | <0.001 | 10.15% (42369/417346) |
| Eye diseases | | 45.05% (149415/331694) | 60.82% (52093/85652) | <0.001 | 48.28% (201508/417346) |
| Ear diseases | | 20.85% (69154/331694) | 25% (21413/85652) | <0.001 | 21.7% (90567/417346) |
| Peripheral vascular diseases | | 3.06% (10135/331694) | 6.95% (5956/85652) | <0.001 | 3.86% (16091/417346) |
| Varicose | | 0.54% (1792/331694) | 1.13% (972/85652) | <0.001 | 0.66% (2764/417346) |
| Hemorrhoid | | 6.11% (20267/331694) | 8.87% (7594/85652) | <0.001 | 6.68% (27861/417346) |
| Congestive heart failure | | 1.3% (4299/331694) | 2.42% (2075/85652) | <0.001 | 1.53% (6374/417346) |
| Chronic obstructive pulmonary disease | | 11.08% (36746/331694) | 18.52% (15867/85652) | <0.001 | 12.61% (52613/417346) |
| Asthma | | 10.78% (35767/331694) | 18.2% (15589/85652) | <0.001 | 12.31% (51356/417346) |
| Bronchiectasis | | 0.91% (3005/331694) | 1.59% (1359/85652) | <0.001 | 1.05% (4364/417346) |
| Lung disease due to external causes | | 0.06% (198/331694) | 0.11% (96/85652) | <0.001 | 0.07% (294/417346) |
| Allergic rhinitis | | 29.9% (99186/331694) | 44.23% (37886/85652) | <0.001 | 32.84% (137072/417346) |
| Chronic rhinitis | | 10.67% (35389/331694) | 14.77% (12649/85652) | <0.001 | 11.51% (48038/417346) |
| Sinusitis | | 7.14% (23693/331694) | 11.17% (9571/85652) | <0.001 | 7.97% (33264/417346) |
| Nasal polyp | | 0.79% (2622/331694) | 1.14% (977/85652) | <0.001 | 0.86% (3599/417346) |
| Throat diseases | | 8.93% (29611/331694) | 14.62% (12519/85652) | <0.001 | 10.09% (42130/417346) |
| Esophagitis | | 14.14% (46891/331694) | 23.67% (20278/85652) | <0.001 | 16.09% (67169/417346) |
| Gastritis | | 56.52% (187485/331694) | 74.41% (63733/85652) | <0.001 | 60.19% (251218/417346) |
| Inflammatory bowel disease | | 0.79% (2621/331694) | 1.13% (969/85652) | <0.001 | 0.86% (3590/417346) |
| Irritable bowel syndrome | | 17.09% (56680/331694) | 26.26% (22495/85652) | <0.001 | 18.97% (79175/417346) |
| Anal diseases | | 1.46% (4853/331694) | 2% (1717/85652) | <0.001 | 1.57% (6570/417346) |
| Alcoholic fatty liver disease | | 1.95% (6480/331694) | 2.75% (2353/85652) | <0.001 | 2.12% (8833/417346) |
| Toxic liver disease | | 0.88% (2908/331694) | 1.4% (1195/85652) | <0.001 | 0.98% (4103/417346) |
| Non-alcoholic fatty liver diseases | | 3.01% (9996/331694) | 5.07% (4345/85652) | <0.001 | 3.44% (14341/417346) |
| Cholecystitis | | 0.26% (872/331694) | 0.45% (383/85652) | <0.001 | 0.3% (1255/417346) |
| Other cholecystic diseases | | 0.11% (366/331694) | 0.24% (208/85652) | <0.001 | 0.14% (574/417346) |
| Biliary diseases | | 0.13% (417/331694) | 0.26% (222/85652) | <0.001 | 0.15% (639/417346) |
| Acute pancreatitis | | 0.26% (877/331694) | 0.47% (402/85652) | <0.001 | 0.31% (1279/417346) |
| Other pancreatic diseases | | 0.2% (679/331694) | 0.42% (364/85652) | <0.001 | 0.25% (1043/417346) |
| Skin diseases | | 48.68% (161477/331694) | 66% (56527/85652) | <0.001 | 52.24% (218004/417346) |
| Musculoskeletal diseases | | 65.09% (215916/331694) | 83.78% (71762/85652) | <0.001 | 68.93% (287678/417346) |
| Urolithiasis | | 2.24% (7429/331694) | 3.4% (2915/85652) | <0.001 | 2.48% (10344/417346) |
| Other urologic diseases | | 20.75% (68842/331694) | 31.58% (27048/85652) | <0.001 | 22.98% (95890/417346) |

For continuous variables, numbers in each cell and parentheses are mean and standard deviation, respectively. BMI=body mass index, BP=blood pressure, AST=aspartate aminotransferase, ALT=alanine aminotransferase, GGT=gamma-glutamyl transferase.

**Supplementary Table 2.** Operational definitions of underlying medical conditions

| Hepatocellular carcinoma | C22.0 AND admission |
| --- | --- |
| Other cancers | (C codes except for C22.0) AND admission |
| Diabetes mellitus | E11-14 AND antidiabetic medication |
| Dyslipidemia | E78 AND lipid-lowering medication |
| Hypertension | (I10-13, I15) AND (admission OR outpatient ≥2) |
| Chronic hepatitis virus infection | B15-19 |
| Human Immunodeficiency virus | B_ |
| Schizophrenic or delusional disorders or mental and behavioral disorders due to psychoactive substance use | F_ |
| Chronic liver disease | K72-74, K70.2-70.4 |
| Stroke | I60-64 AND (admission OR emergency room) AND imaging study |
| Ischemic heart disease | I20-25 AND (admission OR death within 1 month due to cardiovascular diseases OR outpatient visits ≥ 4) |
| Atrial fibrillation | I48 AND (admission OR outpatient ≥2) |
| Gastrointestinal bacterial infection | A01-09 |
| Other bacterial infection | A20-99 |
| Other viral infection | B20-34 |
| Fungal infection | B35-49 |
| Gastrointestinal benign tumors | D12-13 |
| Thyroid benign tumors | D34 |
| Other benign tumors | D_ |
| Blood disorders | D5-8 |
| Hypothyroidism | E00-03 |
| Other thyroid diseases | E04-07 |
| Other endocrine diseases | E2-3 |
| Other metabolic diseases | E79, E8, E9 |
| Mood disorders | F3 |
| Mental disorder due to organic causes | F0 |
| Headache | G43-44 |
| Sleep disorders | G47 |
| Neuropathy | G5-6 |
| Eye diseases | H0-5 |
| Ear diseases | H6-9 |
| Peripheral vascular diseases | I73 |
| Varicose | I83 |
| Hemorrhoid | I84 |
| Congestive heart failure | I50 |
| Chronic obstructive pulmonary disease | J41-44 |
| Asthma | J45-46 |
| Bronchiectasis | J47 |
| Lung disease due to external causes | J60-67 |
| Allergic rhinitis | J30 |
| Chronic rhinitis | J31 |
| Sinusitis | J32 |
| Nasal polyp | J33 |
| Throat diseases | J35-38 |
| Esophagitis | K20-21, K22.1, K22.7 |
| Gastritis | K25-29 |
| Inflammatory bowel disease | K50-51 |
| Irritable bowel syndrome | K58 |
| Anal diseases | K60-61 |
| Alcoholic fatty liver disease | K70.0, K70.1 |
| Toxic liver disease | K71 |
| Non-alcohol fatty liver diseases | K76.0 |
| Cholecystitis | K81 |
| Other cholecystic diseases | K82 |
| Biliary diseases | K83 |
| Acute pancreatitis | K85 |
| Other pancreatic diseases | K86 |
| Skin diseases | All L codes |
| Musculoskeletal diseases | All M codes |
| Chronic renal disease | N18-19 AND (admission OR outpatient ≥2) |
| Urolithiasis | N20-22 |
| Other urologic diseases | N_ |

Codes used here are according to the Korean Standard Classification of Diseases 6^th^ revision (KCD-6) which is based on the International Classification of Diseases 10^th^ revision (ICD-10).

**Supplementary Table 3.** Variance inflation factors (VIFs) of risk factors for hepatocellular carcinoma development in Cox proportional hazard regression. If a variable’s VIF was larger than 2.5 (marked in bold), it was considered significantly correlated with another variable.

| **Variable** | **VIF** | **Variable** | **VIF** | **Variable** | **VIF** |
| --- | --- | --- | --- | --- | --- |
| **AST** | **7.226** | Nasal polyp | 1.165 | Asthma | 1.113 |
| **ALT** | **6.256** | FHx of hypertension | 1.164 | Other metabolic diseases | 1.112 |
| **Diastolic blood pressure** | **2.590** | Other viral infection | 1.161 | Gastrointestinal bacterial infection | 1.109 |
| **Systolic blood pressure** | **2.536** | FHx of cancer | 1.161 | Lung disease due to external causes | 1.107 |
| Residence_urban | 2.220 | Dyslipidemia | 1.158 | Biliary diseases | 1.107 |
| Residence_metropolitan | 2.183 | Thyroid diseases | 1.156 | Smoking | 1.107 |
| GGT | 1.600 | Hemorrhoid | 1.152 | Mental disorder due to organic causes | 1.104 |
| Fasting blood glucose | 1.538 | Urine pH | 1.146 | Congestive heart failure | 1.103 |
| Income_high | 1.503 | Allergic rhinitis | 1.145 | Sleep disorders | 1.103 |
| Sex | 1.475 | Ear diseases | 1.145 | Human Immunodeficiency virus | 1.099 |
| Hemoglobin | 1.431 | Diabetes mellitus | 1.145 | Irritable bowel syndrome | 1.094 |
| Income_middle | 1.428 | Neuropathy | 1.140 | Alcoholic fatty liver disease | 1.092 |
| Overweight | 1.234 | Other endocrine diseases | 1.136 | Anal diseases | 1.087 |
| Exercise | 1.230 | Other bacterial infection | 1.134 | Other cholecystic diseases | 1.085 |
| FHx of liver disease | 1.220 | Headache | 1.133 | Other urologic diseases | 1.074 |
| Obese | 1.211 | FHx of heart disease | 1.131 | Atrial fibrillation | 1.066 |
| Age | 1.207 | Thyroid benign tumors | 1.129 | Other pancreatic diseases | 1.064 |
| Total cholesterol | 1.203 | Other benign tumors | 1.128 | Musculoskeletal diseases | 1.061 |
| Varicose | 1.188 | Throat diseases | 1.127 | Esophagitis | 1.053 |
| Blood disorders | 1.185 | Gastrointestinal benign tumors | 1.125 | Skin diseases | 1.038 |
| Stroke | 1.183 | Urine glucose | 1.124 | Cholecystitis | 1.037 |
| Peripheral vascular diseases | 1.181 | Bronchiectasis | 1.124 | Inflammatory bowel disease | 1.036 |
| Eye diseases | 1.181 | Schizophrenia or substance use | 1.123 | Acute pancreatitis | 1.025 |
| Hypertension | 1.179 | Fungal infection | 1.123 | Non-alcohol fatty liver disease | 1.020 |
| Urine blood | 1.177 | Chronic rhinitis | 1.119 | Toxic liver disease | 1.016 |
| Ischemic heart disease | 1.176 | Chronic liver disease | 1.119 | Chronic renal disease | 1.012 |
| FHx of stroke | 1.172 | Mood disorders | 1.118 | Gastritis | 1.006 |
| Chronic hepatitis virus infection | 1.169 | Sinusitis | 1.117 | Urolithiasis | 1.005 |
| Chronic obstructive pulmonary disease | 1.169 | Urine protein | 1.114 |  |  |
| Alcohol | 1.168 | FHx of diabetes mellitus | 1.114 |  |  |

AST = Aspartate transaminase, ALT = alanine aminotransferase, GGT = gamma-glutamyl transpeptidase, FHx = family history

**Supplementary Table 4.** Frequency of each variable being selected as a significant predictor of hepatocellular carcinoma development in Cox proportional hazard regression using 1,000 bootstrapped training datasets. The variables in bold showed >85% frequencies.

| **Variable** | **Frequency** | **Variable** | **Frequency** |
| --- | --- | --- | --- |
| **Age** | 100% (1,000/1,000) | **Sex** | 100% (1,000/1,000) |
| **Chronic liver disease** | 100% (1,000/1,000) | **gamma-glutamyl transpeptidase** | 100% (1,000/1,000) |
| **Family history of liver disease** | 100% (1,000/1,000) | **Alanine aminotransferase** | 100% (1,000/1,000) |
| **Chronic hepatitis virus infection** | 100% (1,000/1,000) | **Total cholesterol** | 100% (1,000/1,000) |
| **Human Immunodeficiency virus** | 100% (1,000/1,000) | **Dyslipidemia** | 99.1% (991/1,000) |
| **Diabetes mellitus** | 99.1% (991/1,000) | **Schizophrenic or delusional disorders, or mental disorders due to psychoactive substance use** | 98.2% (982/1,000) |
| **Income level** | 88.8% (888/1,000) | **Obesity** | 86.5% (865/1,000) |
| Fungal infection | 83.5% (835/1,000) | Drinking habit | 81.2% (812/1,000) |
| Other urologic diseases | 79.8% (798/1,000) | Esophagitis | 76.9% (769/1,000) |
| Urine protein | 75.6% (756/1,000) | Chronic rhinitis | 74.7% (747/1,000) |
| Urine glucose | 73.0% (730/1,000) | Hemorrhoid | 67.3% (673/1,000) |
| Thyroid diseases | 64.2% (642/1,000) | Irritable bowel syndrome | 61.9% (619/1,000) |
| Non-alcoholic fatty liver diseases | 55.5% (555/1,000) | Alcoholic fatty liver disease | 51.6% (516/1,000) |
| Family history of diabetes mellitus | 46.2% (462/1,000) | Ear diseases | 45.9% (459/1,000) |
| Headache | 41.8% (418/1,000) | Allergic rhinitis | 39.4% (394/1,000) |
| Urolithiasis | 38.4% (384/1,000) | Cholecystitis | 37.4% (374/1,000) |
| Hypertension | 28.6% (286/1,000) | Exercise habit | 28.4% (284/1,000) |
| Other bacterial infection | 28.0% (280/1,000) | Smoking | 22.4% (224/1,000) |
| Gastrointestinal bacterial infection | 21.1% (211/1,000) | Other benign tumors | 20.0% (200/1,000) |
| Other gallbladder diseases | 19.1% (191/1,000) | Lung disease due to external agents | 17.7% (177/1,000) |
| Skin diseases | 16.6% (166/1,000) | Hemoglobin | 13.4% (134/1,000) |
| Mood disorders | 11.9% (119/1,000) | Fasting blood glucose level | 11.8% (118/1,000) |
| Family history of heart disease | 11.3% (113/1,000) | Gastritis | 10.4% (104/1,000) |
| Residence | 10.8% (108/1,000) | Eye diseases | 10.1% (101/1,000) |
| Benign tumors in lower gastrointestinal tract | 9.9% (99/1,000) | Acute pancreatitis | 9.7% (97/1,000) |
| Diseases in throat | 9.5% (95/1,000) | Other viral infection | 9.4% (94/1,000) |
| Varicose | 8.8% (88/1,000) | Other bacterial infection | 8.1% (81/1,000) |
| Toxic liver disease | 7.8% (78/1,000) | Chronic obstructive pulmonary disease | 7.8% (78/1,000) |
| Other biliary diseases | 7.3% (73/1,000) | Neuropathy | 7.2% (72/1,000) |
| Urine pH | 7.0% (70/1,000) | Musculoskeletal diseases | 6.5% (65/1,000) |
| Urine occult blood | 6.1% (61/1,000) | Diseases in anus | 5.8% (58/1,000) |
| Nasal polyps | 5.5% (55/1,000) | Family history of stroke | 5.3% (53/1,000) |
| Other metabolic diseases | 5.3% (53/1,000) | Family history of cancer | 5.2% (52/1,000) |
| Atrial fibrillation | 4.8% (48/1,000) | Blood disorders | 4.6% (46/1,000) |
| Disability | 4.3% (43/1,000) | Bronchiectasis | 3.7% (37/1,000) |
| Sinusitis | 3.2% (32/1,000) | Other pancreatic diseases | 2.8% (28/1,000) |
| Asthma | 2.6% (26/1,000) | Sleep disorders | 2.6% (26/1,000) |
| Ischemic heart disease | 2.6% (26/1,000) |  |  |
